# Development of an Ultraviolet-C Irradiation Room in a Public Portuguese Hospital for Safe Re-Utilization of Personal Protective Respirators

**DOI:** 10.3390/ijerph19084854

**Published:** 2022-04-16

**Authors:** Jorge Padrão, Talita Nicolau, Helena P. Felgueiras, Carla Calçada, Maria Isabel Veiga, Nuno S. Osório, Marcos S. Martins, Nuno Dourado, António Taveira-Gomes, Fernando Ferreira, Andrea Zille

**Affiliations:** 1 Centre for Textile Science and Technology (2C2T), School of Engineering, University of Minho, 4800-058 Guimarães, Portugal; tali_nicolau@hotmail.com (T.N.); helena.felgueiras@2c2t.uminho.pt (H.P.F.); azille@det.uminho.pt (A.Z.); 2 Life and Health Sciences Research Institute (ICVS), School of Medicine, University of Minho, Campus Gualtar, 4710-057 Braga, Portugal; id6520@alunos.uminho.pt (C.C.); mariaveiga@med.uminho.pt (M.I.V.); nosorio@med.uminho.pt (N.S.O.); 3 ICVS/3B’s—PT Government Associate Laboratory, University of Minho, 4806-909 Guimarães, Portugal; 4 Center for MicroElectroMechanics Systems (CMEMS), School of Engineering, University of Minho, 4800-058 Guimarães, Portugal; mmartins@dei.uminho.pt (M.S.M.); nunodourado@dem.uminho.pt (N.D.); 5 LABBELS—Associate Laboratory, 4800-058 Guimarães, Portugal; 6 Department of Surgery, Pedro Hispano Hospital, Local Health Unit Matosinhos (Public-Private Partnerships), 4464-513 Senhora da Hora, Portugal; taveira.gomes@ulsm.min-saude.pt (A.T.-G.); med1873@gmail.com (F.F.); 7 Faculty of Medicine, University of Porto, 4200-319 Porto, Portugal

**Keywords:** UV-C, respirators, masks, SARS-CoV-2, waste mitigation, reutilization

## Abstract

Almost two years have passed since COVID-19 was officially declared a pandemic by the World Health Organization. However, it still holds a tight grasp on the entire human population. Several variants of concern, one after another, have spread throughout the world. The severe acute respiratory syndrome coronavirus 2 (SARS-CoV-2) omicron variant may become the fastest spreading virus in history. Therefore, it is more than evident that the use of personal protective equipment (PPE) will continue to play a pivotal role during the current pandemic. This work depicts an integrative approach attesting to the effectiveness of ultra-violet-C (UV-C) energy density for the sterilization of personal protective equipment, in particular FFP2 respirators used by the health care staff in intensive care units. It is increasingly clear that this approach should not be limited to health care units. Due to the record-breaking spreading rates of SARS-CoV-2, it is apparent that the use of PPE, in particular masks and respirators, will remain a critical tool to mitigate future pandemics. Therefore, similar UV-C disinfecting rooms should be considered for use within institutions and companies and even incorporated within household devices to avoid PPE shortages and, most importantly, to reduce environmental burdens.

## 1. Introduction

Surgical masks and respirators, such as the FFP2 or (K)N95 mask, are critical personal protective equipment (PPE) that protect against respiratory-transmitted infectious diseases [1]. This type of equipment protects its users and other individuals sharing the same environment from droplets suspended in the air or pulverized through breathing, speaking, sneezing, or coughing [2,3,4,5]. The correct use of this PPE has become one of the main strategies for mitigating the spread of the COVID-19 disease by impeding severe acute respiratory syndrome coronavirus 2 (SARS-CoV-2) transmission [6]. Despite massive worldwide vaccination programs (currently, nearly 65% of the world population has received at least one jab), the use of PPE still maintains a critical role in reducing the transmissibility of COVID-19 [7,8]. Due to the importance of masks and respirators as a mitigating tool against COVID-19 transmission, an unprecedented increase in demand for PPE occurred. PPE production and supply chains suffered overwhelming pressure, which led to a global shortage [9,10].

To cope with the lack of PPE, sterilization methods became increasingly popular. Furthermore, users from low-income countries are often required to wear disinfected PPE due to the unmanageable costs of single-use PPE [11]. Alongside these economic considerations, these disinfection and sterilization processes reduce the environmental burden of PPE. The vast majority of masks and respirators contain in their formulation petro-chemical-based materials, such as melt-blown polypropylene (PP), spun-bond PP, and reinforced melt-blown PP [9]. The incorrect disposal of used PPE carries two important consequences: (i) it increases the likelihood that the discarded PPE will become a vector for pathogens, and (ii) it represents an environmental burden, particularly through microplastic generation [12,13]. Countries need an effective waste management policy to deal with these risks. Nevertheless, having an adequate policy is uncommon in most underdeveloped countries. The lack of effective waste management policies implies an increase in poorly managed plastic waste, such as disposing of it in uncontrolled or open dumpsites, resulting in excess plastic waste and the pollution of waterways, streams, rivers, and oceans. Furthermore, the incineration of PPE waste has been reported [14]. Nevertheless, this procedure, independent of the consideration of whether it is or is not associated with energy production, generates non-negligible quantities of extremely toxic carcinogenic and teratogen gases, representing a serious threat to both the environment and public health [14,15]. Worldwide, single-use masks add 1.6 million tons of waste daily, which creates a need for disinfection and sterilization methods [12].

Disinfection and sterilization approaches may become practical tools for users to provide economic and environmental gains. However, these approaches are not universal, and their potential barriers should be accounted for before selection [9]. Therefore, selecting one depends on a combination of factors. Among the most accepted methods, chemical sterilization has been widely used. Some of its options are considered safe in the United States of America [16,17,18,19], even though chemical treatments can also cause allergies in users when chemical residues remain in PPE [20], in addition to relying on specific and expensive equipment [16,17,18,19]. These barriers propelled the search for alternative methods.

Ultra-violet (UV) germicidal activity was first reported in the nineteenth century [21,22]. UV light encompasses three wavelength ranges: 400 to 315 nm (UV-A), 315 to 280 nm (UV-B), and 280 to 200 nm (UV-C). UV-A has been reported to have negligible biocidal action [23]. UV-B exhibits good bactericidal efficacy in doses of approximately 20 mJ cm^−2^. Interestingly, approximately the same inhibition was obtained using just nearly 5 mJ cm^−2^ of UV-C [24]. The application of UV-C is already extensively used for disinfection and sterilization in the food industry [25,26,27] as well as in the treatment of drinking water and wastewater [28,29,30,31], air [32,33], and some medical equipment [9,34,35,36].

UV-C induces damage to the genomes, breaking bonds and forming photodimeric lesions in nucleic acids, such as deoxyribonucleic acid (DNA) and ribonucleic acid (RNA) [37,38]. Nevertheless, the application of UV-C on surgical masks and respirators for the purpose of disinfection has yet to achieve a clear consensus. This is due to several issues that may be summarized as a lack of critical and decisive information about the process’s efficacy, which undermines the trust in its safety. UV-C efficacy is fully dependent on its irradiation; thus, shading will hinder its effectiveness. Moreover, UV-C has a very low penetration depth (nearly 2 μm) [39]. These limitations are exacerbated by the lack of information about how PPE textile architectures affect UV-C efficacy. Furthermore, the adequate irradiation time and dosage of UV-C are undefined [2,9,28,40]. However, UV-C possesses relevant advantages, such as its low cost, yield, ease of use, generation of residual chemical byproducts, and sustainability due to the absence of the use of chemicals or water [9,41]. Ergo, applying this method might prove helpful in dealing with the economic and environmental issues PPE causes [42].

In this case study, FFP2 respirators used by intensive care unit (ICU) health care workers were inoculated with different microorganisms. After inoculation with each microorganism, the FFP2 respirators were exposed to different dosages of UV-C and were compared with non-UV-C-exposed respirators. The inoculated microorganisms used in the first stage of the study included bacteriophage MS2, which is a potential surrogate of SARS-CoV-2, and the bacteria *Staphylococcus aureus* and *Escherichia coli* [43]. In the second stage, SARS-CoV-2 was used to inoculate the FFP2 respirators. The viability of each microorganism was thoroughly analyzed using culture assays (for the MS2 and bacteria) or through the use of highly sensitive reverse transcription quantitative real-time polymerase chain reaction- (RT-qPCR) based methods (for SARS-CoV-2). The results obtained using all the microorganisms, in particular the MS2 and SARS-CoV-2 microorganisms, that were more resistant to UV-C allowed for the adjustment of the UV-C irradiation period of the UV-C disinfection room constructed in a Portuguese public hospital, thus ensuring the complete sterilization of the PPE and medical textiles.

## 2. Materials and Methods

### 2.1. Respirators and Measurement Equipment

The certified FFP2 respirators used by the ICU staff were kindly provided by the Hospital Pedro Hispano, Portugal. The FFP2 respirators comprised five layers: two spun-bond PP layers, followed by a melt-blown PP mesh (most common filtration layer found at the middle layer) and two additional spun-bond PP layers. Microscopy images of the inner and outer layer of the FFP2 masks were obtained using a Leica DM750M coupled with a high-definition digital camera MC170 HD. The UV-C dosage was determined by using a radiometer HD 2102.2 (Delta OHM) equipped with a UV-C probe (LP471UVC, Delta OHM). The UV-C dosage that each sample received was estimated using Equation (1):

(1) $$Q\left(t\right)=\sum_{0}^{t}u\left(t\right)\times \Delta t,\;\Delta t\;=1\;s\;$$

where u(t) corresponds to the instantaneous value of the input variable compared with time t. Three different low-pressure mercury vapor UV-C lamps were used: 30 W, 55 W, and 75 W. All lamps were heated for at least 10 min before each test. During the mapping of the UV-C dosages in the UV-C room at the Hospital Pedro Hispano, the UV-C probe was facing the central column and the wall lamps in each measurement coordinate.

### 2.2. Antiviral and Antibacterial Assays

#### 2.2.1. Virus

The bacteriophage used was the *E. coli* MS2 from American Type Culture Collection (ATCC) 15597-B1. SARS-CoV-2 samples were obtained from excess fresh swab samples diagnosed as SARS-CoV-2 positive through RT-qPCR in the diagnostic laboratory at the University of Minho—Life and Health Sciences Research Institute (ICVS), University of Minho establishment, with the registration number E149026 issued by the Portuguese regulatory body. Experiments with SARS-CoV-2 isolates derived from human nasal swabs were approved by the competent Institutional Review Board, *Comissão de Ética para a Investigação em Ciências da Vida e da Saúde* (CEICVS), with the reference CEICVS008/2022. For SARS-CoV-2 genome degradation assays, the fresh swab clinical sample was diluted to contain approximately 1000–3000 viral copies mL^−1^, considering the quantification cycle (Cq) of the RT-qPCR assay in relation to the commercial standard reference, and EDX SARS-CoV-2 Standard (SKU: COV019, BioRad) containing synthetic RNA transcripts of SARS-CoV-2 envelope (E), nucleocapsid (N), open reading frames (ORF) 1ab, RNA-dependent RNA polymerase (RdRP), and spike (S) genes at 200,000 copies mL^−1^.

#### 2.2.2. Bacteria

*E. coli* ATCC 15597 was used as MS2 host. To assess antibacterial effectiveness, *S. aureus* ATCC 6538 and *E. coli* ATCC 25922 were tested.

#### 2.2.3. Culture Media, Buffers, and Culture Conditions

MS2 bacteriophage was stored, handled, and diluted in ATCC culture medium 271. MS2 host *E. coli* ATCC 15597 was cultured in liquid medium 271 at 37 °C, 120 rpm shaking speed, for at least 4 h and no more than 12 h. In solid 271 medium, MS2 and *E. coli* ATCC 15597 were incubated for approximately 12 h at 37 °C. *S. aureus* ATCC 6538 and *E. coli* ATCC 25922 were cultured in tryptic soy broth (TSB) at 37 °C, 120 rpm shaking speed, for at least 4 h and no more than 12 h. These bacteria were handled and diluted in phosphate buffer saline (PBS) and cultured in tryptic soy agar (TSA) for approximately 12 h at 37 °C.

#### 2.2.4. Antiviral Evaluation

The methodology used to assess the viability of MS2 and SARS-CoV-2 after UV-C exposure consisted of an adaption that consisted of a merge between the American Association of Textile Chemists and Colorists (AATCC) test method 100-TM100 and International Organization for Standardization (ISO) 18184. In brief, an inoculum containing a known titer of plaque-forming units (PFU of MS2 bacteriophage) or genome copies (SARS-CoV-2) was inoculated on the surface of a square piece of an FFP2 respirator (6.25 cm^2^). The inoculum was exposed to a predetermined UV-C dosage, except for the control, which was protected from UV-C light. The initial MS2 bacteriophage and inoculum concentrations analyzed were approximately 1 × 10^7^ PFU mL^−1^ or nearly 1 × 10^12^ PFU mL^−1^ to simulate high titer contamination. Subsequently, each sample was thoroughly vortexed for at least 1 min in ATCC medium 271 with a volume that was 100-fold superior to the inoculum volume. The MS2 viable concentration was estimated through quantification of PFU mL^−1^ through bacteriophage enumeration by double agar overlays, adapted from the protocol described by Azeredo and colleagues [44].

To measure the antiviral activity of the UV-C dosage, a highly sensitive SARS-CoV-2 RT-qPCR assay was conducted as previously developed to evaluate the degradation of SARS-CoV2 deoxyribonucleic acids. After the incubation time, the different membranes incubated with SARS-CoV-2 inoculum were collected in the respective Eppendorf, and 180 µL of RNA-free water were added to each Eppendorf to obtain a final volume of 200 µL. After this, for each condition, the sample virus was inactivated at 60 °C for 10 min. RNA was extracted using NZY Viral RNA Isolation kit (MB40701, NZYTech, Lda, Lisbon, Portugal) and eluted in 40 µL of elution buffer.

The RT-qPCR reactions were performed with 30 µL final volume, in which 10 µL consisted of target RNA and 20 µL of SensiFAST^TM^ Probe No-ROX One-Step Kit (^TM^BioLine Meridian Bioscience, Memphis, TN, USA). The primers and probes used in this work were previously described [45]. The four primers were used at a final concentration of 333 nM each and 84 nM for each SARS-CoV-2 probe. RT-qPCR assays were performed on a QuantStudio™ 6 Pro (Applied Biosystem, Thermo Fisher Scientific, Waltham, MA, USA). SARS-CoV-2 load in the tested samples was calculated using absolute quantification from the Cq standard curve of each viral probe determined with the commercial standard reference, EDX SARS-CoV-2 Standard (SKU: COV019, BioRad, Hercules, CA, USA). Assays were performed using a 75 W UV-C and done at least in triplicate. Percentage of viral elimination (copy number reduction) was calculated using Cq.

The MS2 and SARS-CoV-2 reductions were estimated according to Equations (2) and (3), respectively:

(2) $$\mathrm{Log}\;\mathrm{reduction}\;\left({\mathrm{PFU}\;\mathrm{mL}}^{-1}\right)=\mathrm{Log}\left[\mathrm{control}\;\left({\mathrm{PFU}\;\mathrm{mL}}^{-1}\right)\right]-\mathrm{Log}\left[\mathrm{exposed}\;\left({\mathrm{PFU}\;\mathrm{mL}}^{-1}\right)\right]$$

(3) $$\mathrm{Copy}\;\mathrm{number}\;\mathrm{reduction}\;\left(\%\right)=\frac{\mathrm{control}\;\left({\mathrm{copies}\;\mathrm{mL}}^{-1}\right)-\mathrm{exposed}\;\left({\mathrm{copies}\;\mathrm{mL}}^{-1}\right)}{\mathrm{control}\;\left({\mathrm{copies}\;\mathrm{mL}}^{-1}\right)}\times 100\;$$

#### 2.2.5. Antibacterial Evaluation

An adaptation of AATCC 100 TM 100 was performed to assess the antibacterial efficacy of UV-C in a similar procedure to that of the antiviral evaluation. 6.25 cm^2^ of respirator surface area were inoculated with *S. aureus* or *E. coli*. The concentration of each inoculum was 1 × 10^7^ colony-forming units (CFU) mL^−1^ in 50 µL. The inoculum from the control samples was protected from UV-C irradiation, whereas the samples were exposed to a predetermined UV-C dosage. After 1 h of incubation at 24 °C, the fabrics were immersed in a solution of 100-fold the volume of the inoculum and were thoroughly vortexed for at least 1 min. Afterward, each solution underwent a serial dilution and was plated in TSA.

The reduction was estimated according to Equation (4):

(4) $$\mathrm{Log}\;\mathrm{reduction}\;\left({\mathrm{CFU}\;\mathrm{mL}}^{-1}\right)=\mathrm{Log}\left[\mathrm{control}\;\left({\mathrm{CFU}\;\mathrm{mL}}^{-1}\right)\right]-\mathrm{Log}\left[\mathrm{exposed}\;\left({\mathrm{CFU}\;\mathrm{mL}}^{-1}\right)\right]$$

#### 2.2.6. Software

The data recorded by the UV-C measuring equipment were collected using the DeltaLog9^®^ version 7.30 (Padua, Italy) software. All data were analyzed using Microsoft Office Excel^®^ 2016 version 16.0.4266.1001 (Redmond, WA, USA). Graphics were generated using GraphPad Prism^®^ 6 version 6.01 (San Diego, CA, USA). The blueprint of the UV-C was generated using Sweet Home 3D^®^ version 6.5 (Paris, France), and the contour plot was generated using Origin 9.1. RT-qPCR data were analyzed using Thermo Fisher Scientific Design & Analysis Software, Version: 2.4.3 (Whaltham, MA, USA), including linear regression and absolute quantification analysis.

## 3. Results

**Figure 1:**
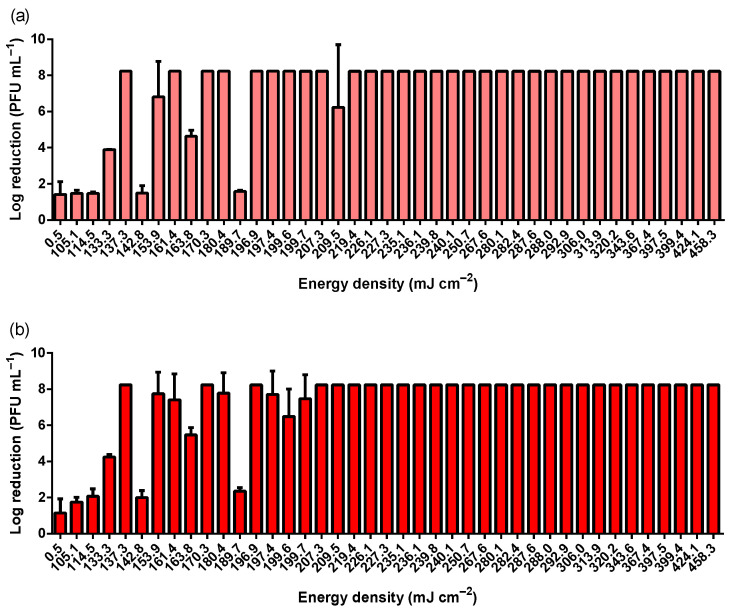
MS2 log reduction according to different UV-C energy densities achieved using two different UV-C lamps (30 and 55 W), different distances between lamp and sample (20, 30, 40, and 50 cm), and different exposure times (5, 6, 7, 8, 9, and 10 min). (**a**) depicts the results obtained on the outer surface of the FFP2 respirator, whereas (**b**) comprises the results achieved at the inner layer of the respirator. MS2 bacteriophage inoculum titer was 1 × 10^12^ PFU mL^−1^.

**Figure 2:**
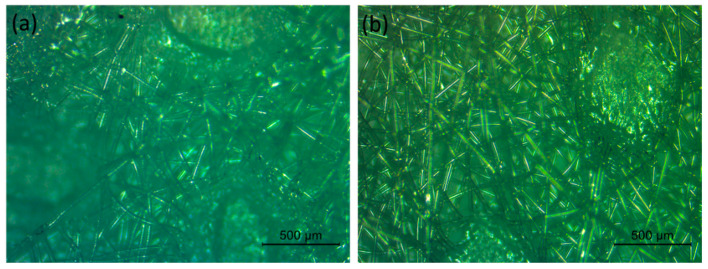
Microscopy images of the (**a**) inner and (**b**) outer layer of an FFP2 respirator.

As a worst-case scenario, a high titer of MS2 bacteriophage (1 × 10^12^ PFU mL^−1^) was used to simulate a considerably high viral contamination concentration. An FFP2 mask contaminated with a high titer of SARS-CoV-2 displayed nearly 1 × 10^6^ PFU mL^−1^ [46]. Figure 1 comprises MS2 log reductions (PFU mL^−1^) exposed to different energy densities. No obvious differences are observed between the outer and inner surfaces of the FFP2 respirator (Figure 2). Despite the high concentration of MS2 particles, UV-C exposures above 209.5 mJ cm^−2^ resulted in a total reduction in the PFU within the dilutions analyzed (above 10^3^). This roughly corresponds to a highly robust viability reduction in approximately Log 8 (PFU mL^−1^) at UV-C dosages higher than 209.5 mJ cm^−2^. These results were obtained within a Class II Biosafety Cabinet. Table 1 depicts, in light grey, the combination of lamp power, time, and distance between the lamp and the sample used to achieve an effective and robust antiviral UV-C dosage on the FFP2 respirator surface.

**Figure 3:**
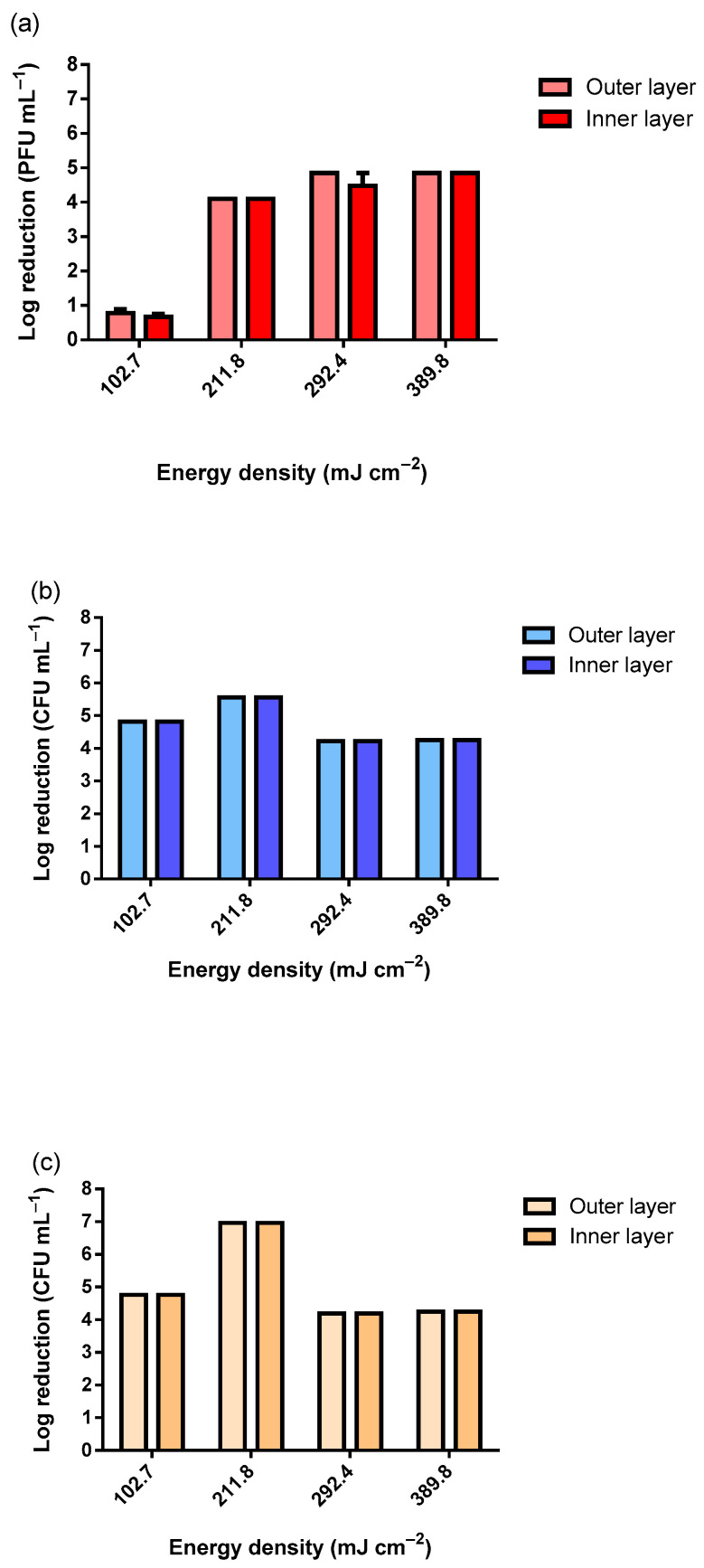
Viability reduction in (**a**) MS2 bacteriophage (inoculum titer: 1 × 10^7^ PFU mL^−1^), (**b**) *S. aureus* and (**c**) *E. coli* (inocula concentration: 1 × 10^7^ CFU mL^−1^) according to the different UV-C dosages using a 75 W UV-C lamp.

After observing a stable reduction in the viability of the MS2 bacteriophage, the experiments were conducted in a room using a UV-C lamp of 75 W, similar to the lamps that would be installed in the hospital room. The defined distance was 50 cm since this was the maximum distance tested in the previous assays, and this was a more operationally feasible distance for the hospital room. Moreover, a more realistic viral titer was tested (1 × 10^7^ PFU mL^−1^). Figure 3 exhibits the viability reduction in MS2 bacteriophage (Figure 3a), *S. aureus* (Figure 3b), and *E. coli* (Figure 3c).

**Figure 4:**
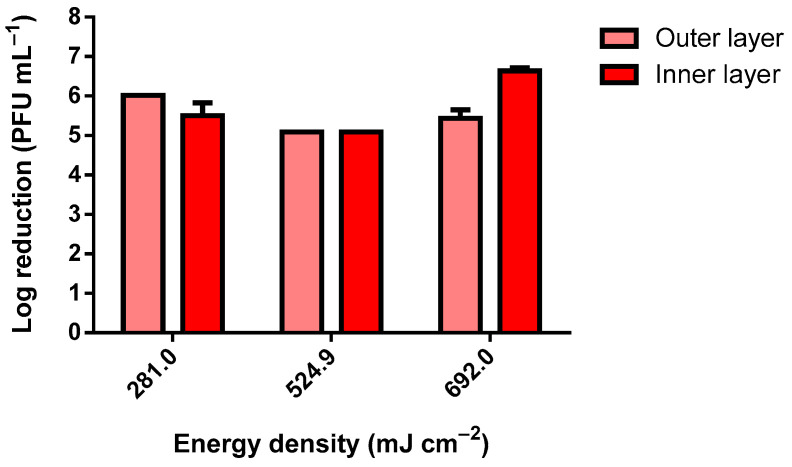
MS2 bacteriophage log reduction using a 75 W UV-C lamp (inoculum titer: approximately 1 × 10^12^ PFU mL^−1^).

As observable in Figure 3, only the lowest tested dosage of 102.7 mJ cm^−2^ did not reduce the viability of the MS2 bacteriophage completely within the tested range. Using 102.7 mJ cm^−2^, the observed reduction was only nearly Log 1 (PFU mL^−1^). As for the tested bacteria, for all UV-C dosages, the reduction was complete within the tested range. The slight differences observed between the samples exposed were due to the slight differences in the inocula concentrations. To simulate the worst-case scenario, the MS2 inoculum with approximately 1 × 10^12^ PFU mL^−1^ was analyzed (Figure 4). The slightly higher reduction at 10 min of UV-C exposure was due to the higher concentration of the inoculum. The results presented in Figure 4 clearly show that even when using an extremely high concentration of MS2 bacteriophage, a dosage higher than 281.0 mJ cm^−2^ in a room at 50 cm from the target is highly effective at reducing its viability.

**Figure 5:**
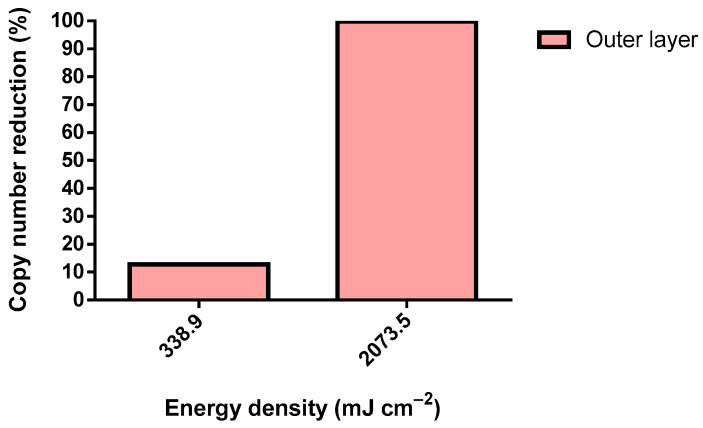
SARS-CoV-2 copy number reduction using a 75 W UV-C lamp (inoculum: >200 copies µL^−1^).

SARS-CoV-2 was exposed to UV-C from a 75 W lamp using similar conditions in a Class III Biosafety Cabinet (Figure 5).

**Figure 6:**
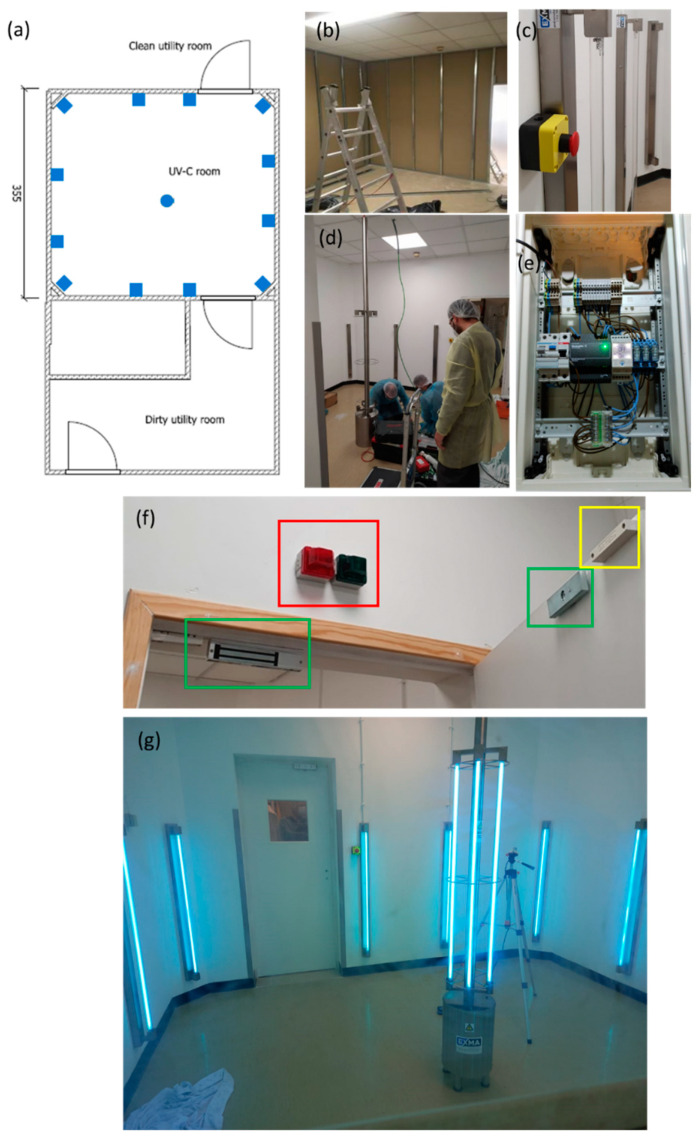
UV-C room. (**a**) blueprint, (**b**) initial construction phase, (**c**) emergency stop button, (**d**) final construction phase (**e**) wireless access to the control cabinet, (**f**) highlighted: in red—warning lights, in green—magnetic lock, in yellow—open/closed door sensor, and (**g**) UV-C room in operation.

A sterilization room was designed and assembled in Hospital Pedro Hispano, Porto, Portugal, within the hospital’s sterilization facilities (Figure 6). The UV-C room comprises a total of sixteen 75 W low mercury pressure UV-C lamps. Twelve lamps were distributed throughout the walls, and four lamps were distributed in a central column. This layout aimed for the maximum minimization of shadowing. The UV-C room was equipped with a wireless control cabinet and several safety systems to prevent the accidental irradiation of operators. These safety measures encompass emergency stop buttons (two buttons inside and two outside the room), operation warning lights, and automatic magnetic door locking systems. The UV-C sterilization cycle will only start if both doors are fully closed (controlled by sensors). When a certified operator (with wireless access to the control cabinet) initiates a cycle, the doors will be magnetically locked automatically, and the door warning lights will switch from green to red. The doors will remain locked throughout the cycle to avoid accidental entry. It is possible at all times to safely confirm visually, through the door windows, if there is anyone inside of the room or if there are any irregular occurrences. Furthermore, the cycle can be promptly interrupted by the emergency stop buttons located outside or inside the room if an accidental or irregular disinfection cycle occurs.

**Figure 7:**
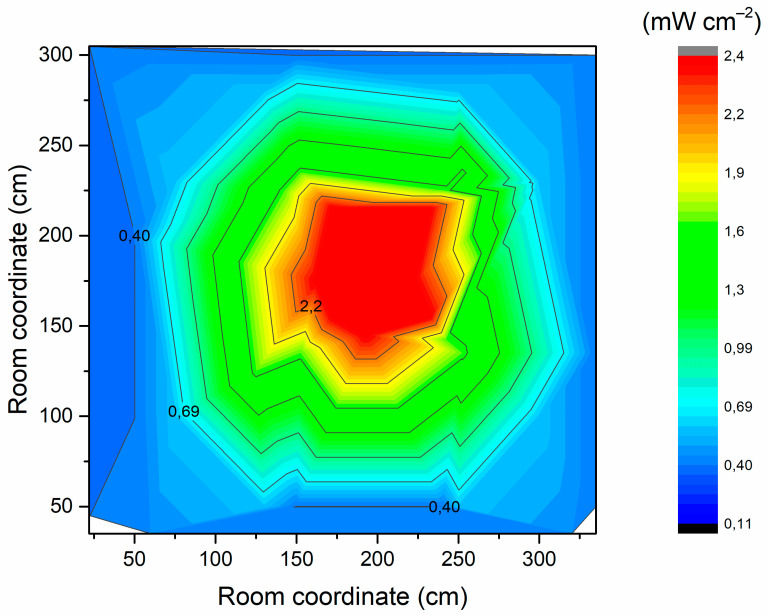
UV-C irradiation inside the room.

The UV-C irradiation of the room was mapped and is displayed in Figure 7.

## 4. Discussion

Several works have focused on the inactivation of MS2 and SARS-CoV-2 through UV-C irradiation [9,47]. Nevertheless, the range of UV-C doses and the effectiveness reported are considerably distinct from each other, most likely due to different experimental setups or quantification protocols, including different inocula concentrations. The results collected here were produced to demonstrate the effective disinfection of FFP2 respirators in a hospital UV-C sterilization room.

The International Ultraviolet Association indicates a range for UV-C to reduce 90% of germ viability, which can be roughly described as 4 mJ cm^−2^ for bacteria, 40 mJ cm^−2^ for fungi, and 45 mJ cm^−2^ for viruses [48]. Thus, as expected, the UV-C was highly effective against *S. aureus* and *E. coli*. No apparent differences were observed regarding UV-C effectiveness between the outer and inner layers of the FFP2 respirator.

MS2 was used due to its potential as a SARS-CoV-2 surrogate and due to its lower biological security level in comparison to SARS-CoV-2, making it a relevant screening tool for testing additional experimental conditions [43].

SARS-CoV-2 was tested only in the outer layer of the FFP2 respirator. Surprisingly, a dosage 1.2-fold higher than the defined dosage threshold reduced only 12.8% of the copy number. Nonetheless, a 7.4-fold higher energy density caused a reduction of 99.8% in the detected viral genome, demonstrating the effective elimination of SARS-CoV-2 through a UV-C irradiation dosage of 2073.5 mJ cm^−2^. Virions require the integrity of several of their structures for adequate host infection. In particular, MS2 comprises (from the core to the extra viral environment) a positive-sense single-stranded (+ss) RNA genome, an assembly of coating proteins (CP) organized in an icosahedral shape, and a single A protein. As for SARS-CoV-2, it is mainly composed of (from the core to the extra viral environment) an (+ss) RNA genome, an N protein that encloses the genome, a virus envelope composed of E proteins, a membrane (M), and finally S proteins [49]. The MS2 A protein and SARS-CoV-2 spike proteins are the most external structures and are responsible for attaching and interacting with the host cell receptor [49,50]. Logically, these proteins could be the first structures to be damaged by UV-C. However, UV-C displays a marginal denaturation of proteins, including the SARS-CoV-2 N and S proteins [51,52]. Therefore, the virucidal activity of UV-C may solely be due to the highly effective pyrimidine dimers that were found within the genome of the exposed organism. Pyrimidine dimers consist of intra-strand covalent bonds located in two adjacent pyrimidines [53]. UV-C is known to cause mainly pyrimidine-pyrimidone (6–4) photoproducts. This damage to genetic material affects single and double-stranded DNA and RNA [53,54]. Pyrimidine dimers completely disrupt the activity of both DNA and RNA polymerase, preventing the replication of microorganisms. N encloses and logically protects SARS-CoV-2 from UV [55]. Therefore, a considerably higher UV-C dose is needed to inactivate SARS-CoV-2 in comparison to MS2. In addition, this underscores the critical importance of preventing shadowing during UV-C treatment.

Furthermore, higher UV-C irradiation may represent a perilous deterioration of the respirator’s properties. However, Lindsay and co-workers observed minor impacts on N95 respirator filtration performance using a dosage of 950,000 mJ cm^−2^ and non-significant effects on the respirator’s structural integrity after exposure to a dosage of 120,000 mJ cm^−2^ [56]. Thus, the dosages of UV-C applied in this work are not expected to have a relevant impact on the respirators; regardless, control tests should be regularly performed.

In Figure 6, the higher irradiation near the central column is clearly observable. It is envisaged that the respirators will be placed within the green zone. Hence, assuming this area is irradiated with 1 mW cm^−2^, only 35 min are required to exceed 2073.5 mJ cm^−2^.

## 5. Conclusions

The COVID-19 pandemic still rages throughout the entire world, and novel variants of concern are persistently swarming. The universal use of surgical masks and respirators has proven to be one of the most effective weapons to prevent infection. Therefore, to cope with their constant demand and to mitigate their waste generation, several disinfecting methodologies have been proposed to reutilize surgical masks and respirators. This study characterizes the UV-C procedure to be followed in a UV-C room constructed in a hospital. The UV-C room has the potential to disinfect masks, respirators, and other equipment through 35 min cycles, although its effectiveness against SARS-CoV-2 must be confirmed and regularly controlled, as well as the damage that the masks and respirators might suffer during irradiation. However, the construction and operation of these sterilization rooms should be planned not only for health care facilities but also for large companies to broaden PPE waste moderation. UV is used in some domestic equipment to diminish the microbial load of, for instance, hearing aids. The same principle should be transferred to masks and respirators as long as a UV-C dosage higher than 2073.5 mJ cm^−2^ is guaranteed. Therefore, the widespread implementation of such facilities or equipment may importantly contribute to our preparedness for future pandemics.

## Figures and Tables

**Table 1:** UV-C energy density according to different exposure periods and distances between the lamp and the sample for the UV-C lamps of 30 W and 55 W in a laminar flow cabinet.

		Exposure Period					
Lamp Power	Distance	5 min	6 min	7 min	8 min	9 min	10 min
		UV-C Energy Density (mJ cm^−2^)					
30 W	20 cm	207.3	248.8	290.2	331.7	373.2	414.6
	30 cm	164.4	197.3	230.2	263.1	296.0	328.9
	40 cm	132.3	158.8	185.3	211.7	238.2	264.7
	50 cm	112.8	135.4	158.0	180.5	203.1	225.7
55 W	20 cm	269.5	323.4	377.3	431.2	485.1	539.0
	30 cm	198.5	238.2	277.8	317.5	357.2	396.9
	40 cm	162.7	195.2	227.8	260.3	292.9	325.4
	50 cm	129.4	155.3	181.1	207.0	232.9	258.8

Notes: Highlighted in light grey are the UV-C dosages above 209.5 mJ cm^−2^ that may be considered the most effective in reducing MS2 bacteriophage viability (stable viability reduction of approximately Log 8 (PFU mL^−1^)).

## Data Availability

Not applicable.

## Abbreviations

+ss	positive sense single stranded
AATCC	Textile Chemists and Colorists
ATCC	American Type Culture Collection
ATCC	American Type Culture Collection
CFU	colony-forming units
CP	coating proteins
Cq	quantification cycle
DNA	deoxyribonucleic acid
E	envelope proteins
ICU	intensive care unit
ISO	International Organization for Standardization (ISO)
M	membrane
N	nucleocapsid proteins
PBS	phosphate buffer saline
PFU	plaque forming units PFU
PP	polypropylene
PPE	personal protective equipment
RNA	ribonucleic acid
RT-qPCR	quantitative real-time polymerase chain reaction
S	spike proteins
SARS-CoV-2	severe acute respiratory syndrome coronavirus 2
TSA	tryptic soy agar
UV	ultra-violet
